# Screening for depressive symptoms in cardiovascular patients at a tertiary centre in Trinidad and Tobago: investigation of correlates in the SAD CAT study

**DOI:** 10.1186/s12888-020-02909-1

**Published:** 2020-10-08

**Authors:** Naveen Seecheran, Cathy-Lee Jagdeo, Rajeev Seecheran, Valmiki Seecheran, Sangeeta Persad, Lakshmipatty Peram, Matthew Evans, Justine Edwards, Sheri Thackoorcharan, Britney Davis, Shari Davis, Barbrianna Dawkins, Anisha Dayaram, Michelle De Freitas, Tsarina Deonarinesingh, Jiovanna Dhanai, Cherelle Didier, Shastri Motilal, Nelleen Baboolal

**Affiliations:** Department of Clinical Medical Sciences, Faculty of Medical Sciences, University of the West Indies, 2nd Floor, Building #67, Eric Williams Medical Sciences Complex, Mt. Hope, Trinidad, St. Augustine, WI Trinidad and Tobago

**Keywords:** Cardiovascular disease, Depression, Patient health Questionnaire-9 (PHQ-9), Screening, Trinidad and Tobago

## Abstract

**Background:**

This study aimed to screen cardiovascular patients for depressive symptoms at a tertiary centre in Trinidad and Tobago; and to determine any significant associations amongst patients’ demographics, comorbidities, and cardiovascular medications with depressive symptoms.

**Methods:**

In this observational, cross-sectional study, patients (*n* = 1203) were randomly selected from the cardiology outpatient clinics at the Eric Williams Medical Sciences Complex. After meeting selection criteria, informed consent was obtained, and patients were administered a case report form, which included the Patient Health Questionnaire-9 (PHQ-9). Descriptive analyses included frequency, percentage and summary statistics. Inferential analyses included 95% confidence intervals (CIs), independent sample t-test, Fisher’s exact test, Chi-square test, and multivariate logistic regression.

**Results:**

The study had a 96% respondent rate, whereby the average age was 62 years old. Slightly less than half were male, and 52.5% were female. Over 90 % of the sample had cardiovascular disease (CVD). One-quarter of the sample had a PHQ-9 score of ≥10, with almost one-fifth having no depressive symptoms. Females, lower levels of education and income were all found to be statistically significant at risk for depressive symptoms (all *p*-values < 0.001). Comorbidities associated with depressive symptoms included hypertension, prior cerebrovascular events, chronic kidney disease, and chronic obstructive pulmonary disease with odds ratios (ORs) and 95% confidence intervals (CIs) of OR 1.988 (CI 1.414–2.797), OR 1.847 (CI 1.251–2.728), OR 1.872 (CI 1.207–2.902) and OR 1.703 (CI 1.009–2.876) respectively. Only the cardiovascular medication of ticagrelor was found to be significantly associated with depressive symptoms (*p*-value < 0.001).

**Conclusions:**

Twenty-five percent of screened cardiovascular patients displayed significant depressive symptoms with a PHQ-9 ≥ 10. This study also highlights the importance of implementing a multidisciplinary approach to managing cardiovascular disease and screening for depressive symptoms in this subpopulation. Further studies are required to validate these findings.

**Trial Registration:**

ClinicalTrials.gov number, NCT03863262. This trial was retrospectively registered on 20th February 2019.

## Background

Cardiovascular disease (CVD), as well as depression, are highly prevalent diseases. They both account for a significant decrease in quality of life (QoL) for the patient and impose a significant socioeconomic burden [1]. There is mounting evidence that depression can be considered an independent risk factor to incur a cardiovascular (CV) event [1]. CVD is the leading cause of mortality worldwide, and this is no different in Trinidad and Tobago, where it accounts for up to 40% of adult mortality annually [2]. Trinidad and Tobago’s economy is chiefly derived from petrochemical energies and, to a minor extent, agriculture, and tourism. The national population is approximately 1.4 million and multi-ethnic with nearly equal representation from South Asian, Caribbean Black, and interracial groups [2, 3].

Depression has skyrocketed from the fourth leading cause of disability to the leading cause globally over the past two decades, according to the World Health Organization (WHO) [4]. It is the most common mental disorder in an ambulatory care setting, with almost one-tenth of patients meeting criteria for major depression. While the literature is replete with studies reporting depression in an outpatient setting, the majority of Trinidadian studies have focused on hospitalized patients and suicide [5]. Depression has an estimated prevalence of nearly 15% amongst patients visiting their family physicians, almost 30% among patients with chronic diseases, and approximately 20% among patients treated for type 2 diabetes mellitus (DM) [5, 6]. Depression is highly coincident in patients with CVD. Approximately one-third to almost one-half of these patients suffer from clinically significant depression, with more than one-fifth of patients fulfilling the criteria for major depressive disorder (MDD) [4]. Coronary artery disease (CAD) appears to be the most common type of CVD associated with depression. Patients with atrial fibrillation (AF), chronic heart failure (CHF), coronary artery bypass grafting surgery (CABG), and cardiac implantable electronic devices (CIEDs) have also been observed to have an elevated risk for depression and MDD [4]. CV patients also possess an increased tendency for other psychiatric illnesses such as generalized anxiety disorder (GAD) and post-traumatic stress disorder (PTSD) [4]. In a previous study, 40% of hospitalized CV patients displayed clinical depression [7]. In another local study, using the Centre for Epidemiologic Studies (CES) Depression scale, almost half of patients with self-reported CVD had depressive symptoms and, conversely, a little more than one-third of patients with depressive symptoms had no self-reported CVD [8].

The pathophysiology of depression is complex and multifactorial; and involves genetic, psychosocial, and neurohumoral factors [9, 10]. Contrarily, the pathophysiology of CVD is relatively better understood. A common implicated pathophysiological mechanism appears to be inflammation; however, it cannot account for the entirety of a causal link between the two conditions [11]. Depression is considered to be a risk factor for developing CVD, as it has attendant effects on endothelial dysfunction, atherosclerotic plaque integrity, the hemostasis-thrombosis axis, and cardiac myocyte electrophysiology [12]. The American Heart Association (AHA) now recommends that depression should be recognized as a risk factor for CVD similar to traditional risk factors such as DM, hypertension (HTN), and dyslipidemia (HLD) [13]. Notably, there is an increased mortality rate in adults with depression with or without CVD [13]. Depression has been shown to have a deleterious impact on patients with CVD with respect to lifestyle, treatment, and compliance [13]. Furthermore, it is associated with a diminished QoL and worsens the overall prognosis [14]. There is a multitude of investigational tools used to assess symptoms of depression, such as the Beck’s Depression Inventory (BDI), Patient Health Questionnaire-2 (PHQ-2), Patient Health Questionnaire-9 (PHQ-9), and the Zung Depression Rating Scale (ZDRS). The PHQ-9 has been shown to have reasonable sensitivity and specificity of almost 90% for MDD in patients with CVD [4, 15, 16]. It is recommended that physicians use tools such as the PHQ-9 to screen patients with CVD for depression as there is a bidirectional link [15, 17–19]. Other key advantages of the PHQ-9 include that it is swift, accurately grades the severity of depression, and can be used to reassess patients on antidepressant therapy [20]. The disadvantages are that it may misdiagnose depression and does not include exclusion criteria for other mood disorders [20].

The principal aim of this study is to screen CVD patients for depressive symptoms at a tertiary center in Trinidad and Tobago; and to determine any significant associations amongst patients’ demographics, comorbidities, and cardiovascular medications with depressive symptoms.

## Methods

### Study design and patient population

The study design was a cross-sectional, descriptive study performed at the Eric Williams Medical Sciences Complex (EWMSC), Trinidad, during the timeframe of December 2018 to June 2019. EWMSC is considered the major cardiovascular tertiary hospital under the North Central Regional Health Authority (NCRHA). The populous of Trinidad and Tobago is approximately 1.4 million people, in which the north-central distribution of the country seeks healthcare at this institution [2]. The primary objectives of the study were to screen patients for depressive symptoms in those with CVD in Trinidad and Tobago and to determine any significant associations amongst patients’ demographics, comorbidities, and cardiovascular medications with depressive symptoms.

### Patient interview and case report form

Patients for the study were recruited from the cardiology outpatient clinics (COPCs) at EWMSC. Patients were screened with a stratified permuted block randomization technique, which applied to recruitment days (weekdays during standard working hours). Randomization sequence numbers were obtained from Statistical Package for Social Sciences (SPSS) version 24.0 software (International Business Machines (IBM) SPSS Statistics, New York, USA). On average, 100 patients were enrolled every week for 3 months. Patients were recruited in the study while waiting to see their clinic physician in a private, confidential consultation room. Once informed consent was obtained from these patients, a case report form (CRF) with the PHQ-9 questionnaire was administered to these patients by the primary investigator. The depression screening tool used in the questionnaire of this study was the PHQ-9. Based on the prior studies, a PHQ-9 ≥ 10 has been noted to have a specificity and sensitivity of 88% for MDD and, thus, was used as a cut-off to represent the percentage of patients with significant depressive symptoms. It was selected primarily because it is user-friendly and used in several similar studies in low-resource settings [21]. This tool also allowed researchers to assess the category of depressive symptoms into mild, moderate, and severe. Patients were enrolled in the study once they fulfilled selection criteria. The inclusion criteria for participation in the study included patients who were officially registered at the COPC for at least 6 months and ≥ 18 years old. The exclusion criteria for participants included unregistered patients, patients < 18 years old, declined participation, inpatients requiring hospitalization, recently discharged patients < 6 months, patients with a previous diagnosis of depression, and patients on antidepressant therapy. No particular gender groups were excluded from this study, nor were any ethnic or racial groups excluded. Illiterate patients were assisted by the clinical research associates.

### Sample size calculation

The final number of enrolled participants in this study was 1203 patients. There were no “dropped” participants, nor were there any incomplete questionnaires by any participants. Based on a study done by Huffman, it was estimated that 40% of patients with CVD also have depression and utilizing a type I error rate of 5% (α = 0.05), a statistical power of 90, 10% patient decline and attrition rate with a minimum detectable difference of 10%, the estimated sample size was calculated to be 1203 patients [4].

### Serious adverse events

Data collection was performed by the principal investigator, who is a licensed practicing physician. The principal investigator was also responsible for the management of any serious adverse events (none occurred), such as an acute cardiovascular or psychiatric condition. Any emergency treatment would be administered at the EWMSC emergency department, located approximately 1–2 min by an attendant-escorted wheelchair. The potential risks associated with the PHQ-9 were low and included psychological trauma, feelings of depression, and acute psychosis. Participants were monitored during the questionnaire by the principal investigator for the emergence of any of the stated potential risks.

### Ethical approval

The principal investigator initially completed a prerequisite ethics course developed by the Collaborative Institutional Training Initiative (CITI) before the application for approval. Full ethical approval was granted by the campus research ethics committee (CREC) of the University of the West Indies, St. Augustine (UWI STA). Additionally, institutional approval was obtained from the Public Health Observatory (PHO) of the NCRHA. The study was registered with the clinical trials registration site ClinicalTrials.gov number, NCT03863262.

Confidentiality of patients was ensured as no patient names were recorded, and all existing data recorded was de-identified. Patients were uniquely identified using their medical record numbers of the COPC. Additionally, the data was only accessible to the primary investigator of the study. The data was stored on a password-protected, encrypted database on a workstation located in a locked office. The scores of the PHQ-9 were verbally communicated to the patients in confidentiality by the primary investigator with the interpretation of the score. Patients were allowed to decide if they wanted the results communicated to their supervising physician in the COPC, and if so, the results were disseminated to their respective physicians who would have then decided on appropriate referrals.

### Statistical analysis

SPSS version 24.0 software (IBM SPSS Statistics, NY, USA) was utilized for data analytics for both descriptive and inferential statistical methods. Descriptive methods included frequency, percentage and summary statistics. Inferential methods included 95% confidence intervals (CIs), independent sample t-test, Fisher’s exact test, Chi-square test, and multivariate logistic regression. Adjusted odds ratios were used to determine the strength of the associations. Statistical significance was accepted as a *p*-value < 0.05.

## Results

### Demographics

Out of 1253 patients approached, 1203 provided informed consent to participate in the study, giving an overall response rate of 96%. The average age of the study population was approximately 62 years old, with the distribution near equivalent for males and females (Table 1). There were no other gender groups. The female population was found to have more depressive symptoms than the male population with a *p*-value of < 0.001 (Table 2). The majority of patients in the study were of South Asian descent, which comprised almost three-fifths of the participants, whereas Caribbean Blacks comprised approximately one-third (Table 1). Chi-square testing revealed no associations between depressive symptoms and ethnicity in this population of patients. Based on the educational background of the study population, one-half of patients had primary school education while a little more than 5% of the population had no education. The income levels of these patients ranged from almost 30% of patients having no income at all, with slightly more than half of the patients being pensioners while 3.1% earned more than TTD 10,000.00 per month (Table 1). Patients with no education and very low-income brackets were found to be statistically significant for depressive symptoms using Fisher’s Exact test.

**Table 1:** Demographics of study participants

Demographics (***n*** = 1203)	Number	Percentage
**Age** (years)	62.5 ± 13.1 (Range)	–
**Gender**		
Male	571	47.5
Female	632	52.5
**Education**		
None	9	6.6
Primary	595	49.5
Secondary	400	33.3
Tertiary	129	10.7
**Income**		
None	339	28.2
Pensioner	631	52.5
$5000 - $10,000 TTD	196	16.3
> $10,000 TTD	37	3.1
**Ethnicity**		
South Asian	690	57.4
Caribbean Black	379	1.5
Interracial and/or other	134	1.1
**Comorbidity**		
CVD (definite)	1085	90.2
USA	612	56.4
NSTE-ACS	244	22.5
STE-ACS	229	21.1
PCI	276	25.4
CABG	414	38.2
HF-rEF	687	63.3
HF-pEF	233	21.5
AF	92	7.6
CIEDs	49	4.1
DM	576	47.9
HTN	860	71.5
HLD	614	51.0
CVE	139	11.6
CKD	101	8.4
COPD	69	5.7
PVD	115	9.6
**Cardiovascular Medications**		
Aspirin	826	68.7
Clopidogrel	382	31.8
Ticagrelor	27	2.2
VKA	112	9.3
DOAC	14	1.2
ACEi	505	42.0
ARB	150	12.5
BB	538	44.7
Statin	644	53.5
MRA	61	5.1
Loop diuretics	211	17.5
Ivabradine	88	7.3
CCB	204	17.0
Nitrates	282	23.4
Trimetazidine	313	26.0
Analgesics	23	1.9

Trinidad and Tobago Dollar (TTD), Cardiovascular disease (CVD), Unstable Angina (USA), Non-ST-segment-Elevation Acute Coronary Syndrome (NSTE-ACS), ST-segment-Elevation Acute Coronary Syndrome (STE-ACS), Status post-Percutaneous Coronary Intervention (PCI), Status post-Coronary Artery Bypass Grafting (CABG), Heart Failure with reduced Ejection Fraction (HF-rEF), Heart Failure with preserved Ejection Fraction (HF-pEF), Atrial Fibrillation (AF), Cardiac Implantable Electronic Devices (CIEDs), Diabetes Mellitus (DM), Hypertension (HTN), Dyslipidemia (HLD), Cerebrovascular Events (CVE), Chronic Kidney Disease (CKD), Chronic Obstructive Pulmonary Disease (COPD), Peripheral Vascular Disease (PVD), Vitamin K antagonists (VKA), Direct Oral Anticoagulants (DOAC), Angiotensin-Converting Enzyme inhibitors (ACEi), Angiotensin Receptor Blockers (ARB), Beta-Blockers (BB), Mineralocorticoid Receptor Antagonist (MRA), Calcium Channel Blockers (CCB)

**Table 2:** Association between demographics and significant depressive symptoms (PHQ-9 ≥ 10)

Demographic	PHQ-9 < 10 (***n*** = 899)	Percentage	PHQ-9 ≥ 10 (***n*** = 304)	Percentage	***p***-value
^a^Age (years) mean ± SD	62.57 ± 13.1	–	60.88 ± 13.1	–	0.052
Gender+					
Male	463	51.5	108	35.5	< 0.001
Female	436	48.5	196	64.5	
Education+					
None	46	5.1	33	10.9	0.001
Primary	432	48.1	163	53.6	0.094
Secondary	317	35.3	83	27.3	0.011
Tertiary	104	11.6	25	8.2	0.109
Income+					
None	221	24.6	118	38.8	< 0.001
Pensioner	490	54.5	141	46.4	0.017
$5000 – $10,000 TTD	154	17.1	42	13.8	0.208
> $10,000 TTD	34	3.8	3	1.0	0.012
Ethnicity~					
South Asian	510	56.7	180	59.2	0.426
Caribbean Black	292	32.5	87	28.6	
Interracial and/or other	97	10.8	37	12.2	

^a^Independent Sample t-test, +Fisher’s Exact test, ~Chi-Square test

Patient Health Questionnaire-9 (PHQ-9), Standard Deviation (SD), Trinidad and Tobago Dollar (TTD)

### Comorbidities

Approximately 90% of these patients had CVD, where one-tenth of these patients were registered patients of the COPCs who were referred for suspected CVD awaiting advanced investigations such as coronary angiography and stress testing. Nearly half of the patients were diabetic and dyslipidemic, and nearly three-quarters of patients were hypertensive (Table 1). The number of diseases was positively correlated with having major depressive symptoms with an odds ratio (OR) of 1.248 (95% CI 1.125–1.384). Additionally, the number of drugs was positively correlated with having major depressive symptoms with an OR of 1.062 (95% CI 1.005–1.123). When adjusted for age, gender, ethnicity, income, and level of education, there were several diseases associated with depressive symptoms in this study. CVD, HTN, CKD, and COPD were all significantly associated with depressive symptoms with ORs and 95% CIs of 1.988 (CI 1.414–2.797), 1.847 (CI 1.251–2.728), 1.872 (CI 1.207–2.902), and 1.703 (CI 1.009–2.876) respectively (Table 3).

**Table 3:** Association between comorbidities and significant depressive symptoms (PHQ-9 ≥ 10)

Comorbidity	Unadjusted OR (95% CI)	***p***- value	Adjusted OR^**a**^ (95% CI)	***p***- value
HTN	1.821 (1.330–2.492)	< 0.001	1.988 (1.414–2.797)	< 0.001
HLD	1.347 (1.037–1.750)	0.026	1.263 (0.965–1.653)	0.055
CVE	1.665 (1.142–2.427)	0.008	1.847 (1.251–2.728)	0.002
CKD	1.896 (1.239–2.901)	0.003	1.872 (1.207–2.902)	0.005
COPD	1.741 (1.046–2.897)	0.033	1.703 (1.009–2.876)	0.046

^a^Adjusted for Age, Gender, Education, and Income, Patient Health Questionnaire-9 (PHQ-9), Odds Ratio (OR), Confidence Interval (CI), Hypertension (HTN), Dyslipidemia (HLD), Cerebrovascular Events (CVE), Chronic Kidney Disease (CKD), Chronic Obstructive Pulmonary Disease (COPD)

### Cardiovascular medications

Almost 70% of the study population was on aspirin, while a little less than one-third were on clopidogrel (Table 1). Approximately 2% of patients were on ticagrelor therapy. Nearly one-tenth of patients were on vitamin K antagonists (VKA), and 1% of patients were on direct oral anticoagulants (DOACs). Just over half of the patients were on a statin. Less than one-fifth of patients were on calcium channel blockers (CCB) as compared to almost half of patients that were on beta-blocker therapy (BB) (Table 1). With respect to medication use, the only significant association between depressive symptoms and medication use was found with ticagrelor with a *p*-value of 0.001 (Table 4). Out of 304 patients with PHQ ≥ 10, approximately 5% were on ticagrelor. There were no statistically significant associations found between depressive symptoms and the use of BB, CCB, ACE inhibitors (ACEi), ARBs, and statins.

**Table 4:** Association between cardiovascular medication use and significant depressive symptoms (PHQ-9 ≥ 10)

Drug	^**a**^PHQ-9 < 10 (***n*** = 899)	Percentage	^**a**^PHQ-9 ≥ 10 (***n*** = 304)	Percentage	***p***-value
Aspirin	606	67.4	220	72.4	0.116
Clopidogrel	291	32.4	91	29.9	0.476
Ticagrelor	12	1.3	15	4.9	0.001
VKA	81	9.0	31	10.2	0.568
DOAC	11	1.2	3	1.0	> 0.99
ACEi	363	40.4	142	46.7	0.060
ARB	113	12.6	37	12.2	0.920
BB	399	44.4	139	45.7	0.689
Statin	473	52.6	171	56.2	0.287
MRA	41	4.6	20	6.6	0.174
Loop diuretic	151	16.8	60	19.7	0.257
Ivabradine	71	7.9	17	5.6	0.204
CCB	149	16.6	55	18.1	0.537
Nitrates	202	22.5	80	26.3	0.183
Trimetazidine	222	24.7	91	29.9	0.082
Analgesics	14	1.6	9	3.0	0.145

^a^Frequency (percentages), Patient Health Questionnaire-9 (PHQ-9), Vitamin K antagonists (VKA), Direct Oral Anticoagulants (DOAC), Angiotensin-Converting Enzyme inhibitor (ACEi), Angiotensin Receptor Blocker (ARB), Beta Blocker (BB), Mineralocorticoid Receptor Antagonist (MRA), Calcium Channel Blocker (CCB)

## Discussion

The categories of depressive symptoms were divided into no depression, minimal depression, mild depression, moderate depression, moderately severe depression, and severe depression based on the PHQ-9 score. Patients with significant depressive symptoms were defined as a patient who had a PHQ-9 score of ≥10. PHQ-9 scores ≥10 would include the categories of moderate depression, moderately severe depression, and severe depression. This cut-off was used as it has a sensitivity and specificity of 88% for detecting MDD and useful in determining the severity of depression [21]. It should also be underscored that the PHQ-9 was not used as a diagnostic tool but as a screening modality for depressive symptoms for which it has a reliable track record [22]. Intrinsically, it possesses high sensitivity and negative predictive value; however, its sensitivity and positive predictive value were low, making it an optimal choice to screen patients for depressive symptoms but not in establishing a diagnosis [23]. In our setting, it was applicable as the patients were willing to participate as the questionnaire only comprised a relatively small number of questions. This made it easy to understand and not very time consuming, hence such a high respondent rate for the study. Also, the PHQ-9 has been proven to be useful across multi-ethnic groups, which included American Blacks, Hispanics, and Caucasians, making it very useful in our cosmopolitan setting [24]. One disadvantage of the PHQ-9 is that it is not a diagnostic tool, and thus if patients score ≥ 10, they need further evaluation for the diagnosis of depression [23]. In this study, it was found that approximately 25% out of 1203 patients had significant depressive symptoms, while only nearly one-fifth of this population had no depressive symptoms. This is an alarming finding and alludes to depression being overlooked as a coincident diagnosis. Of importance, in this study, patients who were found to have a PHQ ≥ 10 were advised to seek further evaluation from a psychiatrist for a more in-depth assessment. In the study, no respondents required emergent admission to the emergency department or psychiatric unit. In a population with CVD, the prevalence of depression is three-fold higher than that of the general population and, thus, was likely under-diagnosed, despite being reported as a risk factor [13]. Those who have depression with CVD have a worse outcome than those who are not depressed [25]. This finding may be supported as patients who are depressed are likely to be non-compliant with therapy for their condition [25]. Thus, it is imperative to screen this population of patients to attenuate CV morbidity and mortality, although few studies have shown that community-based screening for depression improves outcomes [26].

### Demographics

The mean age of the participants was 62 years old, in which female patients were more likely to have significant depressive symptoms as compared to males. This gender disparity is consistent with a multitude of other studies, generally indicating a 1.7-fold higher incidence and prevalence in women [27, 28]. Some postulated causes for this trend include hormonal effects, amongst other issues, such as cultural differences [27]. In this study, 64.5% of females displayed depressive symptoms determined by a PHQ-9 ≥ 10. Both genders were nearly equally represented, with women being more marginally included. Generally, women tend to have internal features of depression, whereas men display external features of depression [27]. There were no significant associations between any particular ethnic group and depressive symptoms found in this study. In comparison, in another study that compared Caucasians, Hispanics, and American Blacks, it was found that depression was more prevalent among the minority groups [29]. Possible explanations for these findings were that the minority groups were more likely to be female, lived alone, had one living parent, and more chronic diseases [29, 30]. Physical health was another variable that impacted the rates of depression amongst different ethnic groups as it was found in England that South Asians were more depressed than Caucasians as a result of lesser physical health status [31]. In another local study assessing hospitalized patients with cardiac disease for depression, no particular ethnic group was significantly more depressed as similarly illustrated in this study [7]. Of note, there is a paucity of local data comparing Caribbean ethnicities and their association with the prevalence of depression. About half the population had only a primary level of education. In contrast, a little more than one-tenth of the population had no formal education, and this subgroup was found to have statistically significant depressive symptoms as compared to other educational levels. It is well recognized that lower levels of education are associated with a higher rate of depression in Westernized societies and are likely attributed to socioeconomic factors [32]. In China, it was found that lower levels of education were also associated with lower socioeconomic status and, consequently, an increased likelihood for the development of MDD in a female population cohort [33]. Conversely, the Trøndelag Health Study (HUNT) study revealed a cumulative protective effect of higher levels of education for depression [34]. Some proposed hypotheses that confer this protective effect include higher education level, satisfactory occupations with higher levels of income, healthier lifestyle practices, and enhanced coping mechanisms with stressors [35]. Therefore, it can be seen that the relationship between low educational levels and depression has been firmly established worldwide. Patients with no income or pensioners were found to have significant depressive symptoms (PHQ-9 ≥ 10). In a systematic review, depression was more prevalent in populations where there is a higher degree of income inequality [36]. Additionally, the European Social Survey 2006/2007 found that income inequality was associated with higher rates of depression [37]. It was similarly found that almost one-third of people with depression were in the lowest income or wealth categories as compared to one-tenth in the higher income brackets [38]. These findings were also replicated in this study. Family or household income seems to have some protective effect against depression, as proposed in another study [39]. Hypothesized theories include two main theories of social causation and social selection [40]. Depression may cause stress, reduced coping mechanisms, and adversity, which may be the etiology of low-income settings and increased risk for the development of mental illness as per the social causation theory [40]. The social selection hypothesis postulates that those with mental illness are more likely to have a reduction in socioeconomic status as a result of possible genetic factors, increased incidence of hospitalizations, and increased rates of unemployment [40].

### Comorbidities

Over 90% of 1203 respondents were reported as having CVD in the sample population. However, the remaining 10% of patients were still registered patients of the COPC at EWMSC with no confirmed cardiac diagnosis at this point of evaluation. This is likely attributed to patients being referred from primary care physicians or other specialty clinics for suspected cardiac disease who required either consultations or further workup with investigations such as coronary angiography and stress testing. They were still included in the sample as an “all-comers” study as they were formally registered patients. Depressive symptoms were prevalent in other disease conditions in this study. These included HTN, CVE, CKD, and COPD after being adjusted for age, gender, income, and educational levels. It was found that both diseases, depression, and HTN can impact on each other [41]. Hence, patients who were depressed were found to have poorly controlled HTN, while patients with HTN were found to have depression [41]. The prevalence of depression in patients with HTN was found to be 26.8% [42]. Unexpectedly, there was no association found between patients with DM and significant depressive symptoms even though some literature has supported this association. In another local study performed in 2013, depression was substantial among patients with DM [43]. This association linking depression and DM was revealed in a Malaysian study and the Madrid Diabetes Study where patients with DM were also found to have depression, most likely as a result of their diabetic complications [44–46] The importance of these associations between depression are other comorbidities lies in the fact that poorly controlled diseases are linked to worse outcomes and result in an overall health economic cost [47]. Likewise, there is a relationship between patients who suffered a CVE and depression. Depression was found to be 1.77-times more common in those patients with a previous CVE after suffering an index event [48]. Patients with CKD have also been found to have varying levels of depression [49]. There have been studies which reported that patients with CKD who have depression are undertreated [50]. Similarly, depression is common in patients with COPD. The prevalence of depression in COPD is approximately 40% [51]. The relationship between COPD and depression also seems to be bidirectional [52].

### Cardiovascular medications

Only ticagrelor had a statistically significant relationship with depressive symptoms. It is important to note that a pertinent finding from the study was that patients on BB therapy did not exhibit any significant depressive symptoms despite it being stigmatized as a common culprit. Some studies refute this BB-induced depression [53]. Similarly, CCBs are common drugs implicated in drug-induced depression. Interestingly, there is some degree of controversy with respect to if CCB therapy is linked to depression and increased suicidal rates. In a Swedish observational study, there was a significant correlation between the use of CCB and suicide after being adjusted for age and sex [54]. In this study, that correlation was not found between the use of CCB and depression. In this study, there was no correlation between statin therapy and depression. However, in a Swedish cohort, it was found that statins, specifically simvastatin, may have some protective effect against depression, especially in patients over the age of 40 years old [55]. The drug ticagrelor is an antiplatelet, a direct-acting inhibitor of adenosine diphosphate (ADP) receptor P2Y12 [56]. In the PLATO trial, out of 9235 patients, approximately 1.1% of patients experienced depression with ticagrelor [56]. When compared to clopidogrel, another antiplatelet agent, the rate of depression was quite similar [56]. 15 of 27 patients taking ticagrelor had a PHQ-9 ≥ 10. This finding may have been related to depression being associated with the CVD for which ticagrelor was initiated. Reports in the WHO database from New Zealand and various other countries may suggest a possible safety concern regarding ticagrelor and depression and suicidality. The safety concern has been investigated, and no link between ticagrelor and depression and no depression nor suicidality was demonstrated [57].

### Strengths of the study

One of the significant strengths of the study was that it was adequately powered and met the pre-specified sample size of 1203 participants. To date, this is the most extensive study with respect to sample size, to screen for depressive symptoms in CVD patients in Trinidad and Tobago. There is also a high study participant response rate, which is typical in a hospital setting. A vital feature of the study was that it was conducted at the EWMSC, which is considered an academic tertiary medical center that specializes in CV care. As a result, it is the only public institution that routinely performs coronary angiography and implants CIEDs. This would improve overall external validity, generalizability, and applicability of the study findings as it encompasses a diverse patient catchment. Additionally, multiple analyses were performed, which were adjusted for age, gender, ethnicity, income, and level of education to mitigate any confounding and interaction effects. An interesting trend or signal was that the unadjusted analyses were similar to the adjusted, suggesting minimal interference from external factors.

### Limitations of the study

The study is of a cross-sectional design and thus only reflects the population’s depressive symptoms within a specific timeframe. There are also inherent flaws associated with an administered questionnaire, such as verifying the extent of truthful responses, alluding to a Hawthorne effect in which individuals modify an aspect of their behavior in response to their awareness of being observed [58]. Finally, the PHQ-9 has not been previously validated in Trinidad as has the BDI.

## Conclusion

Twenty-five percent of CVD patients displayed significant depressive symptoms. The information gleaned from this study can inform national policy to improve screening measures and underscores the imperative need for implementing a multidisciplinary approach to managing CVD. Further comparable studies with national reference data are required to validate these findings.

## Data Availability

The data that support the findings of this study are available from the corresponding author on request.

## Abbreviations

CVD: cardiovascular disease
CV: cardiovascular
MDD: Major depressive disorder
CIED: Cardiac implantable electronic device
GAD: Generalized anxiety disorder
PTSD: Post-traumatic stress disorder
CES: Centre for Epidemiologic Studies
AHA: American Heart Association
AD: Antidepressant
CAD: Coronary artery disease
ACS: Acute Coronary Syndromes
CVE: Cerebrovascular event
T2DM: Type 2 diabetes mellitus
BDI: Beck Depression Inventory
PHQ: Patient Health Questionnaire
ZDRS: Zung Depression Rating Scale
EWMSC: Eric Williams Medical Sciences Complex
NCRHA: North Central Regional Health Authority
COPC: Cardiology outpatient clinic
SPSS: Statistical Package for Social Sciences
IBM: International Business Machines
NY: New York
USA: United States of America
CREC: Campus Research Ethics Committee
CITI: Collaborative Institute Training Initiative
IRB: Institutional Review Board
NLM: National Library of Medicine
NIH: National Institutes of Health
UWI: University of the West Indies
STA: St Augustine
PHO: Public Health Observatory
TTD: Trinidad and Tobago Dollars
ACEI: Angiotensin-converting enzyme inhibitor
ARB: Angiotensin receptor blocker
CI: Confidence interval
CKD: Chronic kidney disease
COPD: Chronic obstructive pulmonary disease
PLATO: PLatelet inhibition And patient ouTcOmes
WHO: World Health Organization
BB: Beta-blocker
CCB: Calcium channel blocker
